# Fast prediction of blood flow in stenosed arteries using machine learning and immersed boundary-lattice Boltzmann method

**DOI:** 10.3389/fphys.2022.953702

**Published:** 2022-08-26

**Authors:** Li Wang, Daoyi Dong, Fang-Bao Tian

**Affiliations:** School of Engineering and Information Technology, University of New South Wales, Canberra, ACT, Australia

**Keywords:** stenosis, immersed boundary-lattice Boltzmann method, machine learning, deep neural network, convolutional neural network

## Abstract

A fast prediction of blood flow in stenosed arteries with a hybrid framework of machine learning and immersed boundary-lattice Boltzmann method (IB–LBM) is presented. The integrated framework incorporates the immersed boundary method for its excellent capability in handling complex boundaries, the multi-relaxation-time LBM for its efficient modelling for unsteady flows and the deep neural network (DNN) for its high efficiency in artificial learning. Specifically, the stenosed artery is modelled by a channel for two-dimensional (2D) cases or a tube for three-dimensional (3D) cases with a stenosis approximated by a fifth-order polynomial. An IB–LBM is adopted to obtain the training data for the DNN which is constructed to generate an approximate model for the fast flow prediction. In the DNN, the inputs are the characteristic parameters of the stenosis and fluid node coordinates, and the outputs are the mean velocity and pressure at each node. To characterise complex stenosis, a convolutional neural network (CNN) is built to extract the stenosis properties by using the data generated by the aforementioned polynomial. Both 2D and 3D cases (including 3D asymmetrical case) are constructed and examined to demonstrate the effectiveness of the proposed method. Once the DNN model is trained, the prediction efficiency of blood flow in stenosed arteries is much higher compared with the direct computational fluid dynamics simulations. The proposed method has a potential for applications in clinical diagnosis and treatment where the real-time modelling results are desired.

## Introduction

Coronary artery disease is one of the major health threats in the 21st century, and is reported as one of the leading causes of death worldwide (World Health Organization, 2014). This disease, where a localised accumulation of plaque in the arteries surrounding the heart, prevents sufficient blood supply to the heart muscle, can lead to ischemia, stroke, heart attacks, and ultimately death. The hemodynamics of the blood flow has long been thought to play an important role in the pathogenesis and pathophysiology of atherosclerosis (Saxena et al., 2019).

The cardiovascular system typically features low Reynolds number pulsatile flow due to the cyclic pumping motion of the heart. Computational fluid dynamics (CFD) has proven to be an effective method to uncover the fluid dynamics of blood flow. For example, Tu et al. (1992) studied the Newtonian blood flow through a stenosis by using finite element simulations focusing on the effects of various degrees of stenosis, stricture length, Reynolds number and Womersley number. Köhler et al. (2001) compared the results of blood flow in the realistic model of the human carotid bifurcation from CFD simulations with that from magnetic resonance imaging (MRI) measurements finding that CFD is a reliable tool for the flow prediction. A detailed numerical study of the blood flow through a localized stenosis in an idealized 2D blood vessel was conducted by Tian et al. (2013). Abuouf et al. (2020) studied the effects of guidewire position on the measurements of fractional flow reserve (FFR) by using CFD. In addition, the fluid-structure interaction of collapse tubes has been considered as a model of blood-induced deformation of arteries (see e.g., Tang et al. (2015); Wang et al. (2021)). However, the high fidelity CFD simulations are usually time consuming, especially for 3D arterial flows involving complex geometries, making CFD impractical for the clinical diagnosis and treatment where the real-time modelling results are required. To address this challenge, an efficient tool without the assistance of professional fluid dynamics knowledge is desired, which is the focus of this paper.

Among several techniques to provide prediction of blood flow, machine learning (ML) is a promising method which has drawn considerable attentions recently in the field of CFD. It has been successfully applied to solve a series of physical problems. For example, the data-driven machine learning algorithm has been adopted for turbulent flow modelling to obtain a closure subgrid scale stress model for the large eddy simulation or Reynolds-averaged Navier-Stokes equations (Wang et al., 2018; Zhu et al., 2019; Sun et al., 2022). A convolutional neural network was introduced by Mao et al. (2018) to predict the unsteady wave forces on bluff bodies due to the free-surface wave motion. Bukka et al. (2021) presented a recurrent neural network to construct a reduced-order model for the unsteady flow field and fluid-structure interaction. An ML method based on convolutional neural netwrok (CNN) was proposed by Jing et al. to predict the unsteady velocity field around a circular cylinder from the pressure coefficients measurements (Jin et al., 2018). Sekar et al. (2019) presented a data-driven approach by using the combination of CNN and multilayer perceptron for the prediction of laminar flow around NACA airfoil, which achieves the flow prediction of various airfoils. ML has also been combined with CFD tools to study fish swimming (Zhu et al., 2021, 2022). Moreover, ML has been studied in the field of biomedicine, such as prediction of malaria (Lee et al., 2020) and patient quality-of-life after prostate radiation therapy (Yang et al., 2020). An ML based model was applied to the diagnosis of coronary artery diseases by using the fraction flow reserve, without providing the detailed information of the flow field (Coenen et al., 2018). A physics-informed neural network was introduced by Kissas et al. (2020) to solve conservation laws in graph topologies to predict arterial blood pressure from non-invasive unsteady flow MRI data. Husso et al. (2021) introduced an ML to estimate myocardial blood flows from tissue impulse response signal in an animal model. A physical-informed neural netw006Frk was used by Arzani et al. (2021) to obtain the near-wall hemodynamics and wall shear stress data from sparse velocity measurements and without knowledge of the inlet/outlet boundary conditions. These applications inspire us to create a highly efficient predicting tool for the accurate and detailed flow dynamics prediction in the stenosed arteries by using machine learning and CFD, which can be used not only for diseases diagnosis, but also for the treatment. The comprehensive details of the flow properties provide more robust impressions of the disease and therefore better treatment strategies. Deep neural network (DNN) has shown powerful capability in various applications (e.g., LeCun et al. (2015); Ren et al. (2018); Liang et al. (2020)). Compared with the popular reduced-order method such as proper orthogonal decomposition (Guibert et al., 2014) and dynamic mode decomposition (Habibi et al., 2020), a DNN is expected to provide higher fidelity results due to their powerful training capability which could be comparable with high-fidelity CFD simulations. In this work, we explore the powerful capability of DNN for fast predicting the blood flows in stenosed arteries following several relevant studies (Coenen et al., 2018; Liang et al., 2020; Arzani et al., 2021; Husso et al., 2021).

This paper aims at providing accurate and detailed flow properties in stenosed arteries by using an ML, which can be used for both the stenosis diagnosis and design of treatment strategies. To construct the predicting tool based on the ML, an idealised function, which could be arbitrary options with stochastic disturbance, is adopted to generate a large number of stenosis profiles. The learning datasets, the mean flow fields of the constructed stenosed arteries are obtained by using an immersed boundary-lattice Boltzmann method (IB-LBM) (Tian et al., 2011; Wang and Tian, 2018; Xu et al., 2018; Ma et al., 2020) which incorporates the immersed boundary method for its excellent capability in handling complex boundaries and the LBM for its efficient modelling for unsteady flows (Wang et al., 2022). Finally, a DNN is trained and tested for the fast prediction of the mean blood flow in stenosed arteries. Inspired by the CNN-based feature extraction of various airfoils (Sekar et al., 2019), a CNN is also built to extract the stenosis features, which is used to represent the complex stenosis as the input of the DNN. To the best of our knowledge, this is the first attempt to predict the comprehensive flow properties in stenosed arteries by using a DNN model with the IB-LBM.

The rest of this paper is organised as follows: the ML method and IB-LBM used in this study are presented in Section 2; both 2D and 3D DNNs are trained and tested, with the results and discussions being presented in Section 3; final conclusions are given in Section 4.

## Hybrid framework of machine learning and immersed boundary-lattice Boltzmann method

The present approach includes three main parts, i.e., the characterisation of the stenosed artery, the IB-LBM solver for the high-fidelity CFD simulations of the blood flows in stenosed arteries, and the DNN for predicting the mean flow fields. Specifically, the stenosis is first characterised by either the polynomial factors or the CNN extracted features; then high-fidelity CFD simulations are conducted by using the IB-LBM solver to obtain datasets, and finally an approximate DNN is trained by using these datasets.

### Characterization of the stenosed artery

**FIGURE 1:**
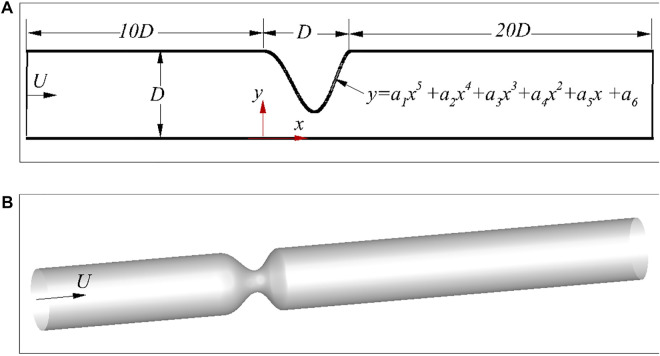
Schematic of the stenosed arteries: **(A)** 2D asymmetric case, and **(B)** 3D axis-symmetric case.

Stenosis is usually caused by the accumulation of lipids in the intima of artery, and it could have different shapes for various patients and arteries (Varghese et al., 2007a,b; Huang et al., 2020). Here, a fifth-order polynomial is used to describe the stenosis, i.e,

$$y=a_{1}x^{5}+a_{2}x^{4}+a_{3}x^{3}+a_{4}x^{2}+a_{5}x+a_{6},$$ (1)

with *y* (0) = *y*(*D*) = *D*, *y*′(0) = *y*′(*D*) = 0 and *D* being the diameter of the artery at the inlet, as shown in Figure 1. The free parameters in Eq. 1 are randomly selected to generate a database for the learning algorithm. Both axis-symmetric and asymmetric 3D arteries are considered here. The axis-symmetric artery has a similar stenosis governed by Eq. 1 as shown in Figure 1, so that the 3D stenosis shares the same characteristic parameters with its 2D counterpart. The asymmetric arteries will be described later in. For simplicity, *D* is used as the length of the stenosis. Although Eq. 1 is an idealised model, these free parameters can be arbitrary options with stochastic disturbance. Therefore, the idealised model does not affect the effectiveness of the method in patient-specific cases which could be modelled with more parameters.

**FIGURE 2:**
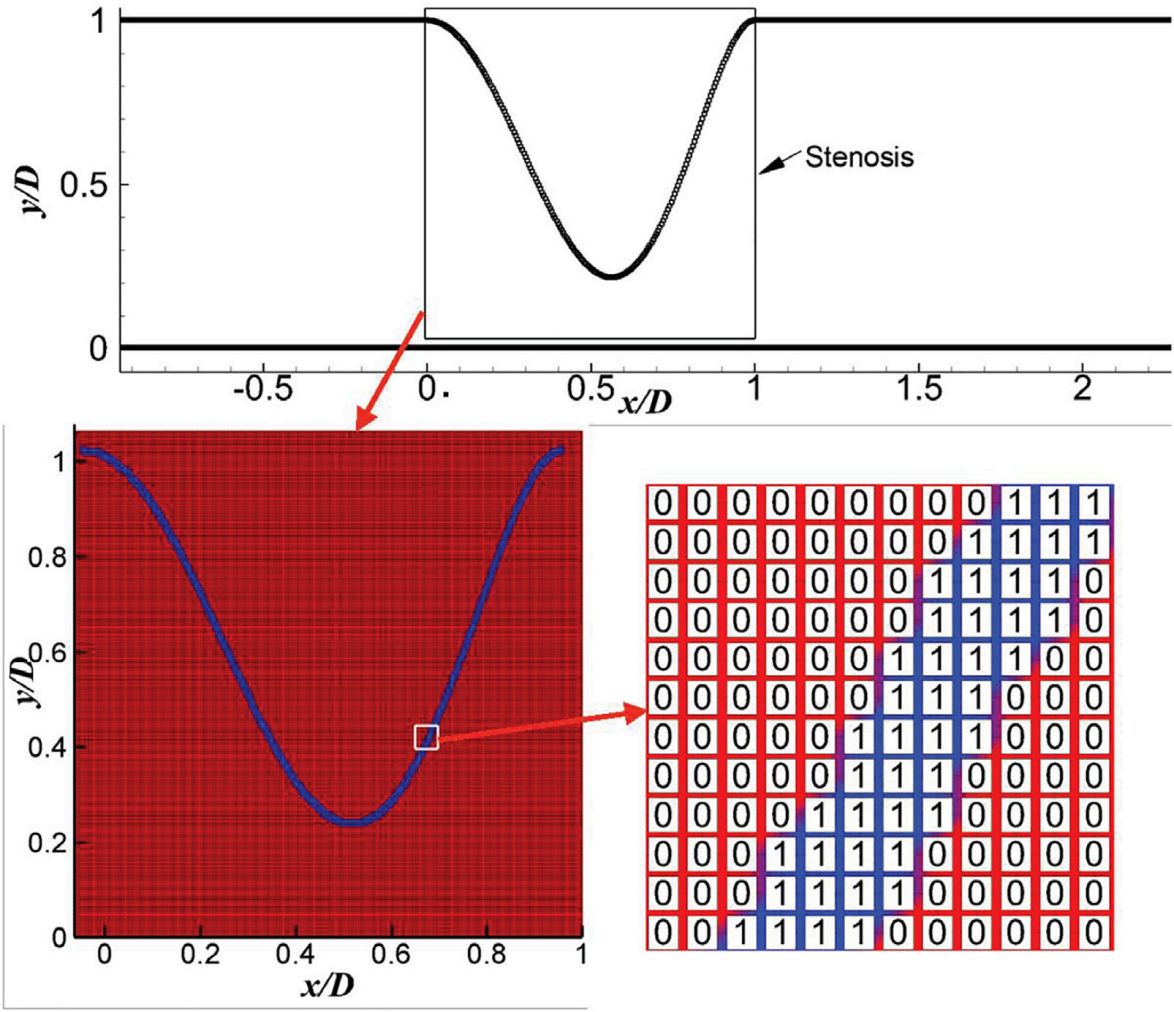
An example of image generation for the input of the 2D CNN.

To represent the stenosis shape, both the coordinates of the stenosis and the free parameters in Eq. 1 can be used as the input of the DNN for the mean flow prediction. However, the large number of stenosis coordinates will definitely make the DNN more complex. There are only six factors in Eq. 1, which seems to be a good representation of the stenosis, and will be discussed later. As the realistic stenosis could be even more complex, a CNN (Sekar et al., 2019) is also built to extract the geometric features of stenosis. Here, the CNN will be trained based on the dataset generated by using Eq. 1, which can be further extended for an arbitrarily complex stenosis. The current CNN includes an input layer, 3 convolutional layers, 5 fully connected layers and an output layer. Rectified linear unit (ReLU) and the hyperbolic tangent functions are used as the activation functions for the convolutional and fully connected layers, respectively. The parameters of the CNN are listed in Table 1. In the 2D CNN, the inputs are 2D images (an example is shown in Figure 2), which is generated by filling the pixels crossed by the stenosis as one with the rest of the pixels as zero, and each image has a resolution of 214 × 214. Here, only the stenosis section of the artery is transferred into the image as the rest part of the artery is uniform. The convolutional layers and the first three fully-connected layers are used to decode the training images into 16 parameters representing the stenosis features, while the rest fully connected layers then encode the features to obtain the *y*-coordinates of the stenosis. The loss function of the CNN is defined as

$$y_{\mathrm{loss}}=\frac{1}{N}\Sigma {\left(y_{\mathrm{pred}}-y_{\mathrm{label}}\right)}^{2},$$ (2)

where *N* is the sample number, and *y*_*pred*_ and *y*_*label*_ are the predicted and labelled y-coordinates, respectively. Further details of the CNN can be found in Refs. (Lee and You, 2019; Sekar et al., 2019; Hasegawa et al., 2020; Bukka et al., 2021).

**TABLE 1:** Details of the 2D CNN.

Layer	Size	Filter size	Pooling filter	Output shape
Input	214 × 214 × 1	-	-	-
Conv1	32	4 × 4	3 × 3	214 × 214 × 32
Conv2	64	4 × 4	3 × 3	71 × 71 × 64
Conv3	128	4 × 4	3 × 3	23 × 23 × 128
Fully Connected × 2	128	-	-	128
Fully Connected (extracted features)	16	-	-	16
Fully Connected × 2	128	-	-	128
Output	-	-	-	201

### Immersed boundary-lattice Boltzmann method solver for the computational fluid dynamics simulation

The flows in the stenosed artery are solved by using an IB–LBM (Tian et al., 2011; Wang and Tian, 2018; Xu et al., 2018; Ma et al., 2020). In this method, the fluid dynamics is solved by the LBM, and the complex no-slip boundary conditions on the fluid–structure interface are achieved by an immersed boundary (IB) method. Without loss of generality, the 2D numerical method is briefly reviewed in this section, and more details of this method and its 3D version can be found in Refs. (Tian et al., 2011; Wang and Tian, 2018; Xu et al., 2018; Ma et al., 2020). The main reason of choosing IB–LBM is due to its high computational efficiency and simplicity in handling arbitrarily complex geometries, which are especially desirable for the large data requirements in ML and more complex stenosed arteries.

In the multiple relaxation time (MRT)-based IB-LBM, the evolution equation of the particle distribution function *g*_*i*_ along the *i*-th direction at position ***x*** is expressed as (He and Luo, 1997; Guo and Zheng, 2008)

$$g_{i}\left(x+e_{i}\Delta t,t+\Delta t\right)=g_{i}\left(x,t\right)-\Omega_{i}\left(x,t\right)+\Delta tG_{i},$$ (3)

where *i* = 0, 1, … , 8, Δ*t* is the time step, ***e***_*i*_ is the lattice speed, Ω_*i*_ is the collision operator, and *G*_*i*_ represents the body force effects on the distribution function. Ω_*i*_ and *G*_*i*_ are defined as

$$\Omega_{i}=-{\left(M^{-1}SM\right)}_{ij}\left[g_{j}\left(x,t\right)-g_{j}^{eq}\left(x,t\right)\right],$$ (4)

$$G_{i}={\left[M^{-1}\left(I-\frac{S}{2}\right)M\right]}_{ij}F_{j},$$ (5)

Where *M* is a 9, ×, 9 transform matrix for the two dimensional nine-speed (D2Q9) model, and *S* is a non-negative diagonal matrix. The details for the determination of *S* and *M* can be found in Ref. (Peng et al., 2006). The lattice speed ***e***_*i*_ in 2D simulation is defined as

$$e_{i}=\left\{\begin{array}{cc} \left(0,0\right),\; & i=0\\ \left(\cos\left(0.5\pi \left(i-1\right)\right),\sin\left(0.5\pi \left(i-1\right)\right)\right)\frac{\Delta x}{\Delta t},\; & i=1,2,3,4\\ \sqrt{2}\left(\cos\left(0.5\pi \left(i-4.5\right)\right),\sin\left(0.5\pi \left(i-4.5\right)\right)\right)\frac{\Delta x}{\Delta t},\; & i=5,6,7,8 \end{array}\right.$$ (6)

where Δ*x* is the lattice spacing. The macro density and momentum are given as follows,

$$\rho =\overset{8}{\underset{i=0}{\sum }}g_{i},\;\rho u=\overset{8}{\underset{i=0}{\sum }}g_{i}e_{i}+\frac{1}{2}f\Delta t.$$ (7)

The local equilibrium distribution function $g_{i}^{eq}$ and the force term *F*_*i*_ are calculated by

$$g_{i}^{eq}=\omega_{i}\rho \left[1+\frac{e_{i}\cdot u}{c_{s}^{2}}+\frac{uu:\left(e_{i}e_{i}-c_{s}^{2}I\right)}{{2c}_{s}^{4}}\right]$$ (8)

$$F_{i}=\omega_{i}\left[\frac{e_{i}-u}{c_{s}^{2}}+\frac{e_{i}\cdot u}{c_{s}^{4}}e_{i}\right]\cdot f$$ (9)

where the sound speed $c_{s}=\Delta x/\left(\sqrt{3}\Delta t\right)$ , ***f*** is the force acting on the fluid, and the weights *ω*_*i*_ are given by *ω*_0_ = 4/9, *ω*_*i*_ = 1/9 for *i* = 1, 2, 3, 4 and *ω*_*i*_ = 1/36 for *i* = 5, 6, 7, 8 in 2D domain. The relaxation time *τ* (one of the components of $S$) is related to the kinematic viscosity *ν* of the fluid according to $\nu =\left(\tau -0.5\right)c_{s}^{2}\Delta t$ . The non-equilibrium extrapolation method is used for the boundary conditions at the out boundaries of the computational domain (Guo et al., 2002). For 3D simulations, D3Q19 MRT-LBM is used (Wang and Tian, 2018). The feedback IB method (Goldstein et al., 1993; Kim and Peskin, 2007; Tian et al., 2011; Huang and Tian, 2019) is used to handle the no-slip boundaries between the structure and fluid. The IB method is a type of Cartesian-mesh-based methods which has excellent capability in handling complex boundaries (Peskin, 2002; Mittal and Iaccarino, 2005; Tian et al., 2012; Huang and Tian, 2019). In this work, the Newtonian fluid flow in 2D and 3D arteries is considered at a Reynolds number (Re = *U*_0_ *D*/*ν*, where *D* being the diameter of the artery and *U*_0_ being the average incoming velocity) of 100. It should be noted that this Reynolds number is considered, as it captures the major flow features in arteries, while the computational cost is low. In addition, the steady uniform velocity boundary condition is applied at the inlet. This is different from the realistic arterial flows that are pulsatile. However, the conclusions obtained can be extended to cases of higher Reynolds numbers and pulsatile inlet boundary conditions which would generate more complicated flow structures such as secondary vortex flows and turbulence.

**FIGURE 3:**
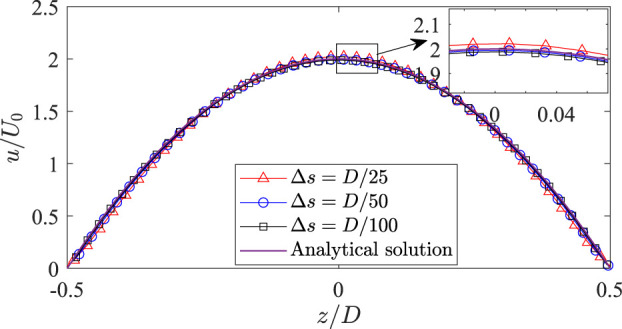
Velocity profile in a 3D cylindrical pipe measured at a distance of 5*D* from the inlet.

The IB-LBM solver and its previous version used here have been validated in our previous work extensively considering fluid–structure interaction in steady and unsteady flows (Wang and Tian, 2018; Xu et al., 2018, 2019; Wang and Tian, 2019; Ma et al., 2020; Huang et al., 2021; Wang, 2021). To further validate the IB-LBM solver in modelling internal flows (e.g., hemodynamics with immersed structures), a Newtonian flow in a 3D cylindrical pipe is conducted. In this problem, a constant inlet velocity (*U*_0_) is applied on the left side of the computational domain. The fluid domain has a dimension of 10*D* × 0.6*D* × 0.6*D*. A mesh convergence study is conducted at a Reynolds numbers of 100 with three mesh spacings of the fluid domain, i.e., Δ*s* = *D*/25, *D*/50 and *D*/100. The mesh spacing of the cylinder is roughly half of the fluid mesh spacing. The velocity profiles at a distance of 5*D* from the inlet are shown in Figure 3 along with the analytical solution

$$u=2U_{0}\left(1-\frac{4r^{2}}{D^{2}}\right),$$ (10)

where, *r* is the distance to the center of the pipe. As shown in Figure 3, the velocity profiles calculated by the present numerical method agree well with the exact solutions in Eq. 10. It shows that the numerical results are in excellent agreement with the analytical solutions when the mesh is refined to *D*/50. Note that the velocity profile is corrected by using *D* + 0.5Δ*x* in the IB-LBM simulations, with Δ*x* being the mesh spacing of the fluid domain containing the structure, as suggested in Refs. (Huang et al., 2020, 2021).

### Deep neural network for the flow prediction

**FIGURE 4:**
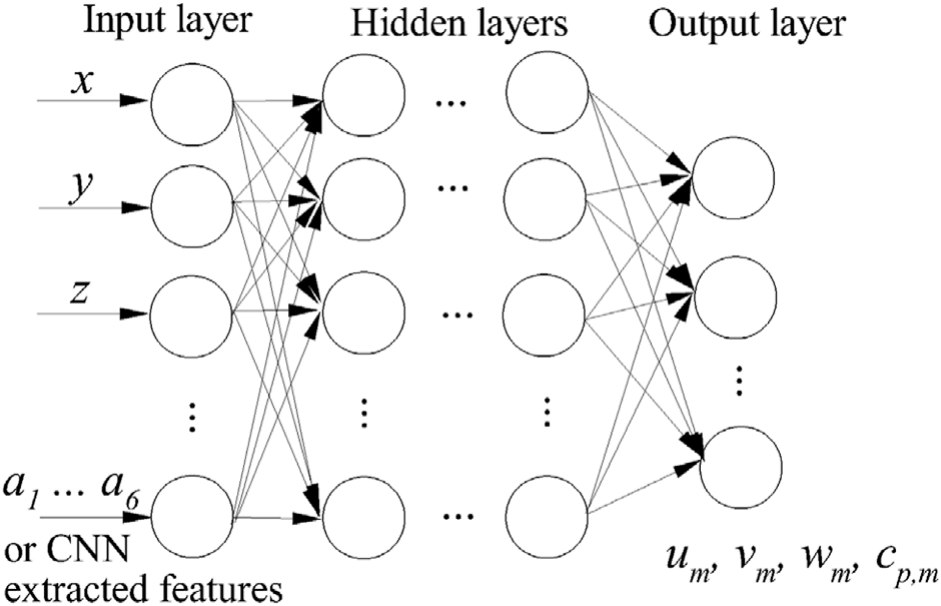
Architecture of the DNN.

Once the characteristic parameters of the stenosis and the mean flow fields (calculated over a dimensionless time of 50 after the initial flow developing periods) are obtained, a DNN is then constructed to generate an approximate model for the fast prediction of the mean flow field close to the stenosis, i.e., from *D* upstream and 4*D* downstream of the stenosis. The architecture of the DNN used for the flow field prediction is shown in Figure 4. It consists of an input layer, 5 hidden layers (each of them has 128 perceptrons) and an output layer. Here, the inputs are the stenosis characteristic parameters (i.e., the free factors of Eq. 1 or the characteristic parameters extracted by the CNN) and the coordinates of fluid nodes, and the outputs are the mean flow velocities (*u*_*m*_, *v*_*m*_ and *w*_*m*_) and pressure (*c*_*p*,*m*_) at each fluid node (the output shape is 3 for 2D and 4 for 3D), while the output in Ref. (Liang et al., 2020) is the values of the whole flow field and thus the output shape is significantly larger and varies with the stenosis size. By using such an architecture, the DNN will have significantly less trainable parameters due to the smaller output shape and thus may be more efficient in the training stage. Similarly to the CNN, the root-mean-square error is defined as the loss function for the DNN. The ReLU function is adopted as the activation function as it is less susceptible to vanishing gradients that prevent deep models from being trained compared with other functions such as Sigmoid and Tanh. The adaptive moment estimation (Adam) is used as the optimizer. During the training process, back propagation method is used to update the trainable parameters in the NN according to the gradients of the loss function. All the NNs in this study are created by using the open source library Tensorflow (Abadi et al., 2015) because of its simple implementations.

The major procedures of the present approach to predict the blood flow are as follows:

1) Generate stenosis samples by using Eq. 1, and the stenosis is then characterised by using the free parameters of Eq. 1;
2) For the stenosis characterised by using CNN-extracted features, the 2D images are first generated according to the stenosis coordinates. The network is trained to get a model for the extraction of the stenosis features by feeding these image samples to the CNN and training;
3) Conduct high fidelity CFD simulations of the randomly selected samples to generate a dataset for the DNN;
4) Organise the dataset with the input being fluid node coordinates and stenosis features, and the output being the mean flow velocity and pressure.
5) Feed the organised dataset to the DNN and train the network to obtain the approximate model for the fast prediction of the blood flow field.

When the clinical patient-specific stenosis data are available, it can be modelled by more parameters and fed into the CNN instead of the data generated from the polynomial for the feature extraction. It means that the current framework by combining the CNN and DNN can be still effective for the patient-specific geometries. Another important reason to use CNN for the stenosis feature extraction is because the arteries are usually constructed by using MRI which can be directly used as the input of the CNN.

## Results and discussions

### 2D flow prediction in a channel with a stenosis

**FIGURE 5:**
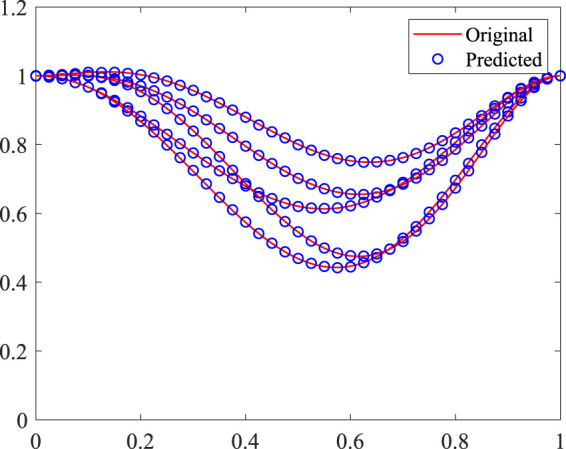
Comparison of the predicted and original y-coordinates of 2D stenosis.

By feeding the image dataset generated by using Eq. 1 into the CNN to characterise the arteries, a trained model can be obtained. Here, 1,000 samples are used as the training dataset and 200 samples are used as the validation dataset. The learning rate is set as 1.0, ×, 10^−4^. Some validation samples are shown in Figure 5. It is found that the predicted y-coordinates of the stenosis agree well with the original data, which confirms that the CNN is accurate in characterising the stenosis.

**FIGURE 6:**
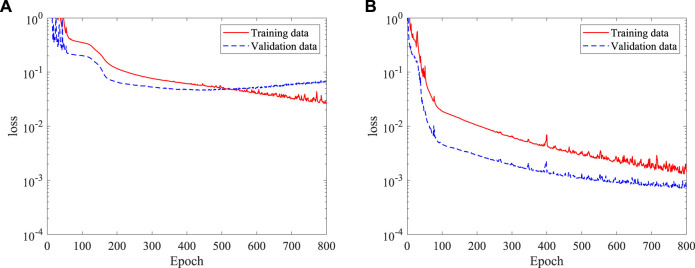
Time histories of loss of the DNN in a 2D channel with a stenosis: **(A)** polynomial factors, and **(B)** CNN-extracted characteristic parameters.

Here, 100 CFD simulations are conducted to obtain the training dataset for the DNN to predict the blood flow, with another 20 simulations as the validation dataset. There are approximately 150,000 data points in each simulation, and 1.5 × 10^7^ data points in total are used to train the DNN. The initial learning rate is set as 1.0, ×, 10^−3^, and the learning rate is gradually decreased to around 4.0, ×, 10^−5^ in order to stabilize the learning process. Two DNNs are trained by using the polynomial factors and the characteristic parameters extracted by the CNN, respectively. Figure 6 shows the time histories of the loss from the two DNNs. It is found that the DNN using features extracted by the CNN achieves a significantly smaller loss compared that using the polynomial factors. This indicates that the CNN has a better ability in representing the stenosis features. As a general approach, it also can be extended to arbitrarily complex stenosis with the required dataset such as MRI or CT images obtained in clinic practices.

**FIGURE 7:**
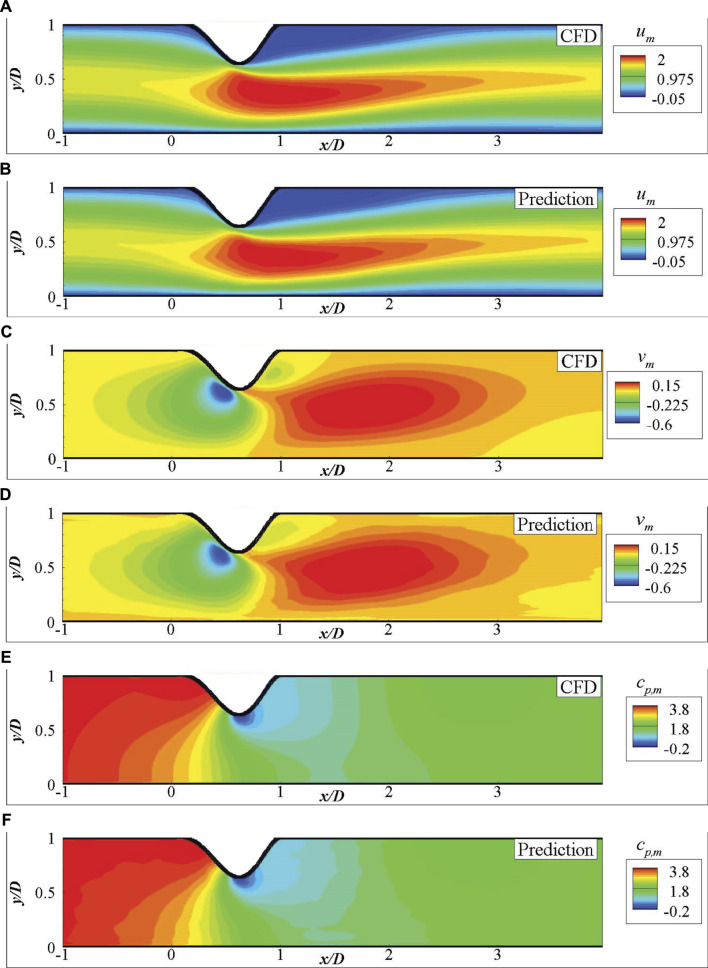
Comparison of the mean fluid fields for 2D channel flows from DNN prediction and CFD simulation: **(A)** *u*_*m*_ by CFD, **(B)** *u*_*m*_ by DNN prediction, **(C)** *v*_*m*_ by CFD, **(D)** *v*_*m*_ by DNN prediction, **(E)** *c*_*p*,*m*_ by CFD, **(F)** *c*_*p*,*m*_ by DNN prediction.

**FIGURE 8:**
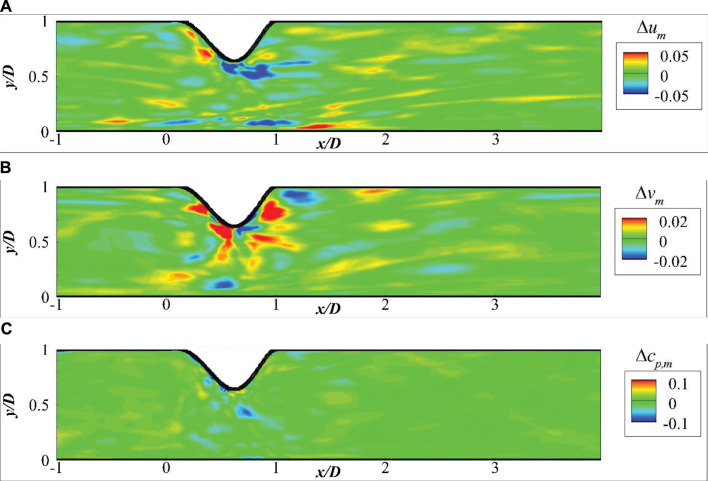
Absolute errors of the mean fluid field: **(A)** Δ*u*_*m*_, **(B)** Δ*v*_*m*_, and **(C)** Δ*c*_*p*,*m*_.

**FIGURE 9:**
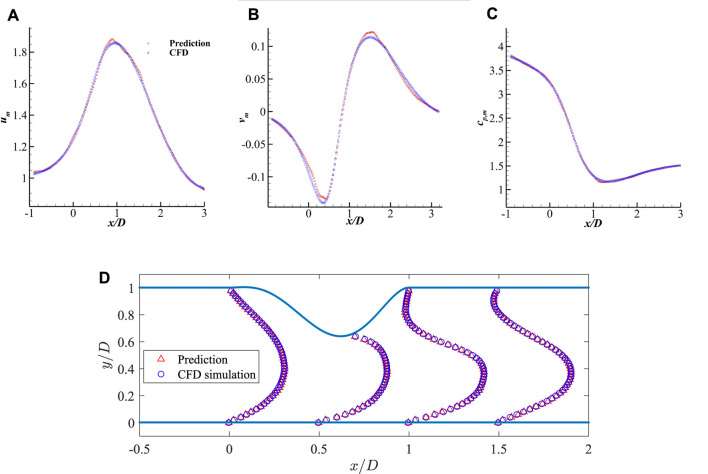
Comparison of the mean velocities and pressure along the streamwise direction measured at *y* = 0.2*D* and the comparison of streamwise velocity profile: **(A)** *u*_*m*_ along x-axis, **(B)** *v*_*m*_ along x-axis, **(C)** *c*_*p*,*m*_ along x-axis, and **(D)** streamwise velocity profile *u*_*m*_ along y-axis.

The mean flow fields (i.e., $u_{m}=\bar{u}/U$ , $v_{m}=\bar{v}/U$ and $c_{p,m}=\bar{p}/\left(0.5\rho U^{2}\right)$ ) of one validation by using the CNN extracted features are presented in Figure 7, which shows that the predictions from the DNN is very close to the high fidelity CFD simulations. The mean fluid field errors are further shown in Figure 8, which demonstrates that the error of the predictions is mostly observed close to the wall due to the drastic changes. The local error is less than 5% of the maximum velocity near the centre, acceptable in such fast predictions. A more robust comparison of the mean velocities and pressure measured along the streamwise direction at *y* = 0.2*D* are presented in Figure 9, which shows a good agreement of the predicted fluid values and those from CFD simulations with acceptable errors.

### 3D flow prediction in axis-symmetric artery

**FIGURE 10:**
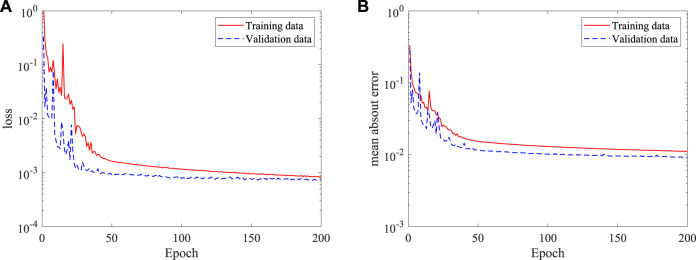
Histories of loss and mean absolute error of the DNN for 3D (axis-symmetric) flow prediction: **(A)** loss, and **(B)** mean absolute error.

To demonstrate the ability of the DNN in considering more realistic blood flows, the DNN model is further trained based on 3D simulation data to predict the blood flow. A similar DNN architecture of the 2D case is adopted for the 3D model, except that the input and output have an extra dimension. By feeding 100 3D simulation data, around 1.6 × 10^8^ data points, to the DNN, an approximate model is obtained by using Adams optimizer. Figure 10 shows the time histories of the loss and mean absolute error, it is found that the training approaches to a small loss after training for 200 epochs, the mean absolute error is also small compared with the absolute value of dimensionless velocities and pressure (generally in a range more than 1.0).

**FIGURE 11:**
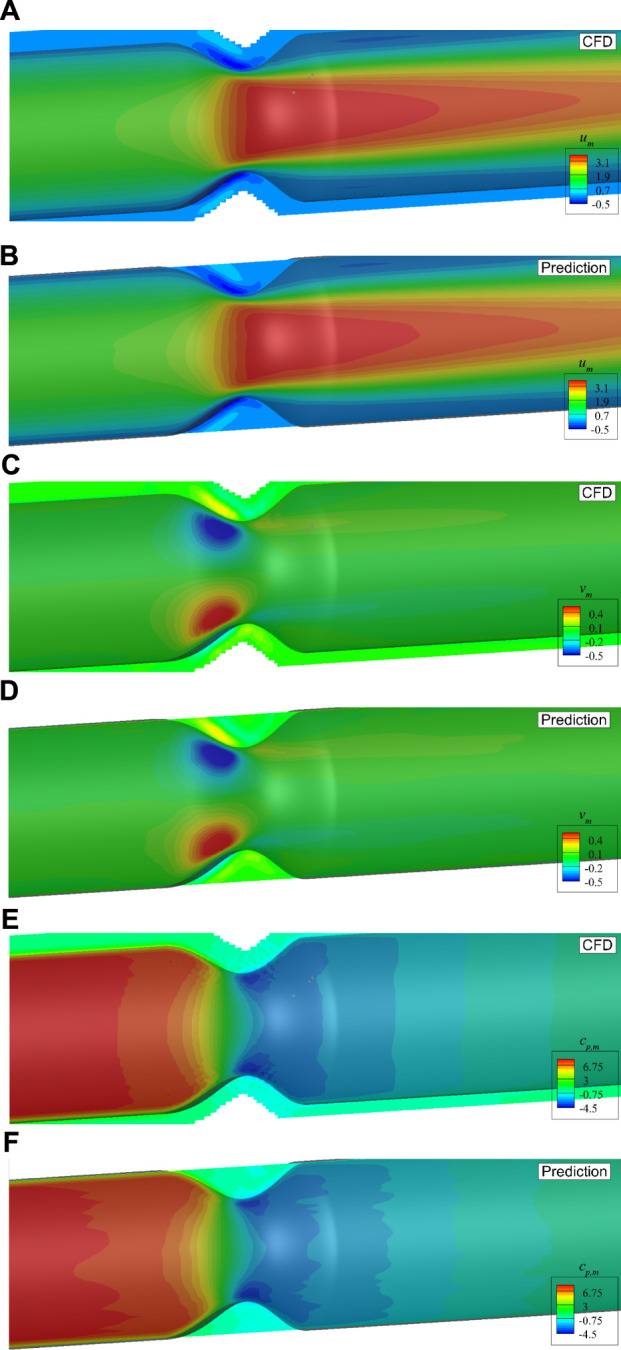
Comparison of the mean velocities and pressure with axis-symmetric stenosis on the plane of *z* = 0: **(A)** *u*_*m*_ by CFD, **(B)** *u*_*m*_ by DNN prediction, **(C)** *v*_*m*_ by CFD, **(D)** *v*_*m*_ by DNN prediction, **(E)** *c*_*p*,*m*_ by CFD, **(F)** *c*_*p*,*m*_ by DNN prediction.

**FIGURE 12:**
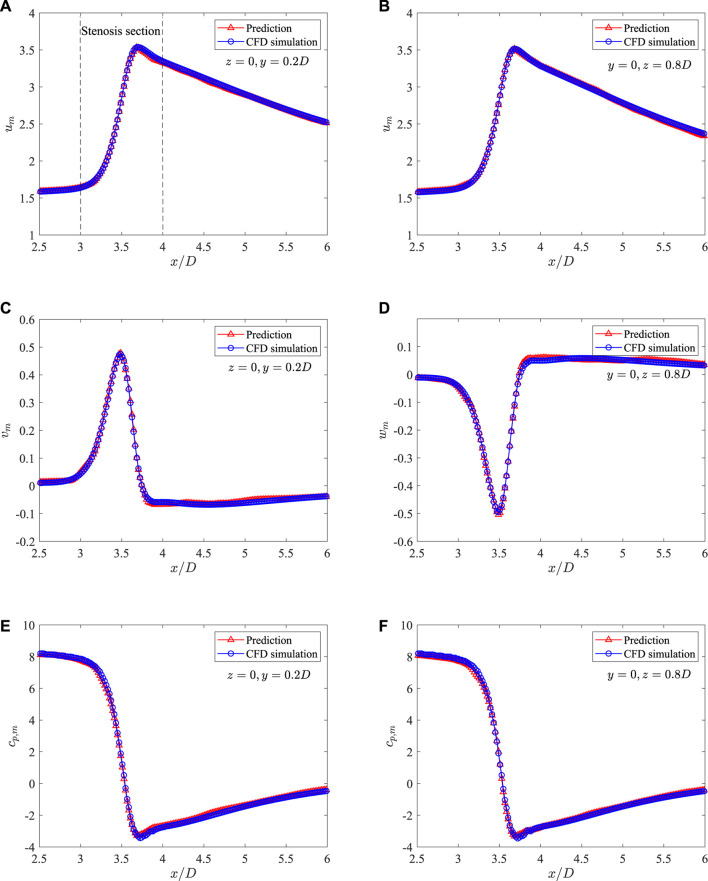
Comparison of the mean velocities and pressure along the streamwise direction measured on two lines in the 3D axis-symmetric case: **(A)** *u*_*m*_ at *z* = 0 and *y* = 0.2*D*, **(B)** *u*_*m*_ at *z* = 0 and *y* = 0.8*D*, **(C)** *v*_*m*_ at *z* = 0 and *y* = 0.2*D*, **(D)** *v*_*m*_ at *z* = 0 and *y* = 0.8*D*, **(E)** *c*_*p*,*m*_ at *z* = 0 and *y* = 0.2*D*, and **(F)** *c*_*p*,*m*_ at *z* = 0 and *y* = 0.8*D*.

Figure 11 shows a direct comparison of the mean flow fields from DNN prediction and CFD simulation on the plane of *z* = 0. It is found that the predicted flow fields are very close to those from high fidelity CFD simulation. The stenosis effects on the velocity and pressure change are well captured by the DNN. A more careful comparison of the dimensionless velocities and pressure distributions in the streamwise direction measured on two lines, i.e., line 1 with *z* = 0 and *y* = 0.2*D*, and line 2 with *y* = 0 and *z* = 0.8*D*, is presented in Figure 12. It shows negligible discrepancies and further confirms the accuracy of the present machine learning approach.

### 3D flow prediction in asymmetric artery

**FIGURE 13:**
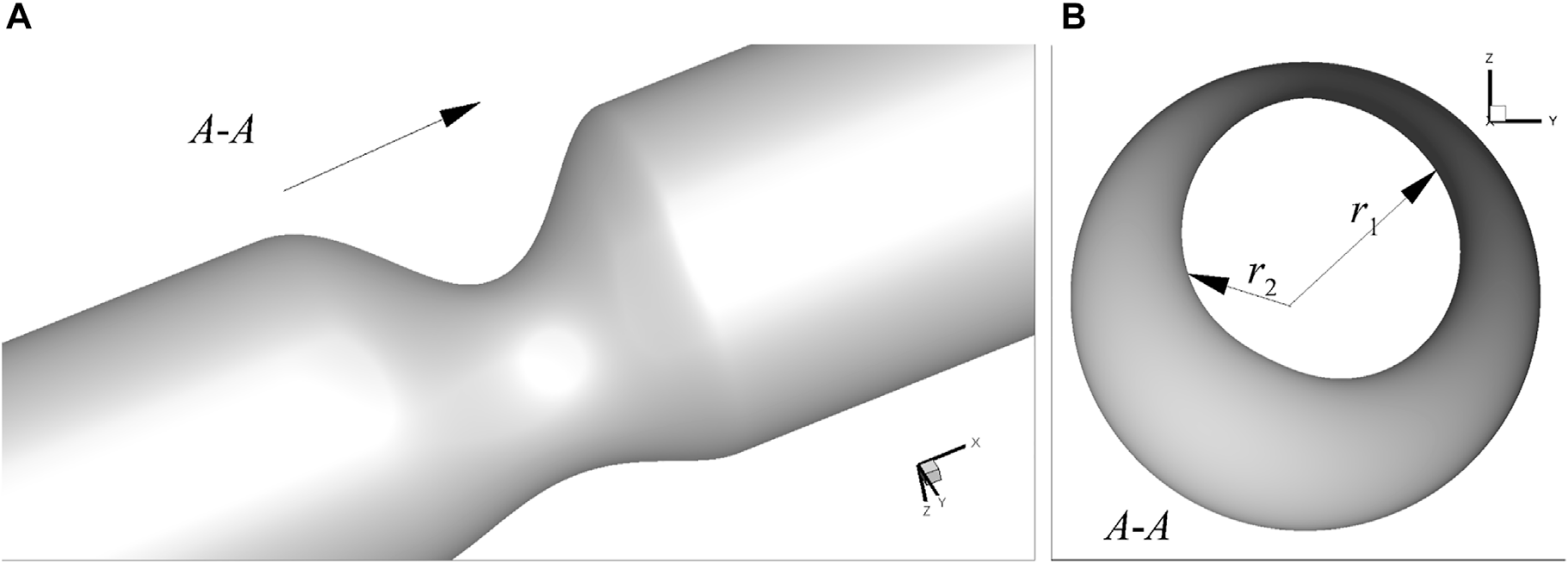
An example of 3D asymmetric stenosed artery: **(A)** 3D view, and **(B)** *x*-axis view.

The 3D flow in axis-symmetric artery presented in the last section shows that the present method has good ability to predict the flow properties in such stenosed arteries. However, the real stenosed arteries are generally not symmetric, and thus the simplified axis-symmetric model may not be applicable. Here, to clarify the ability of the present method in predicting flow properties in more practical stenosed arteries, we use the combination of Eq. 1 and a normal distribution function to describe the radius along the circumferential direction at the stenosed section. Therefore, the stenosis with complex and asymmetric shape can be represented. An example is shown in Figure 13 to illustrate the asymmetric stenosed arteries generated by this model.

**FIGURE 14:**
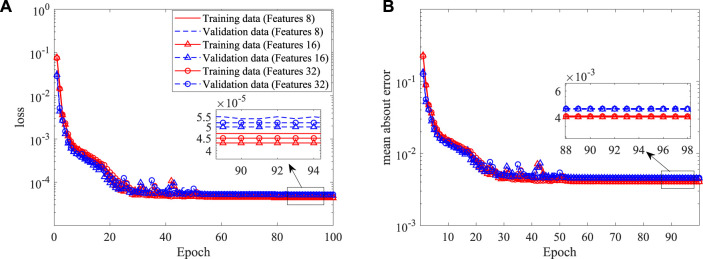
Histories of loss and mean absolute error in the shape construction of the 3D CNN: **(A)** loss, and **(B)** mean absolute error.

**FIGURE 15:**
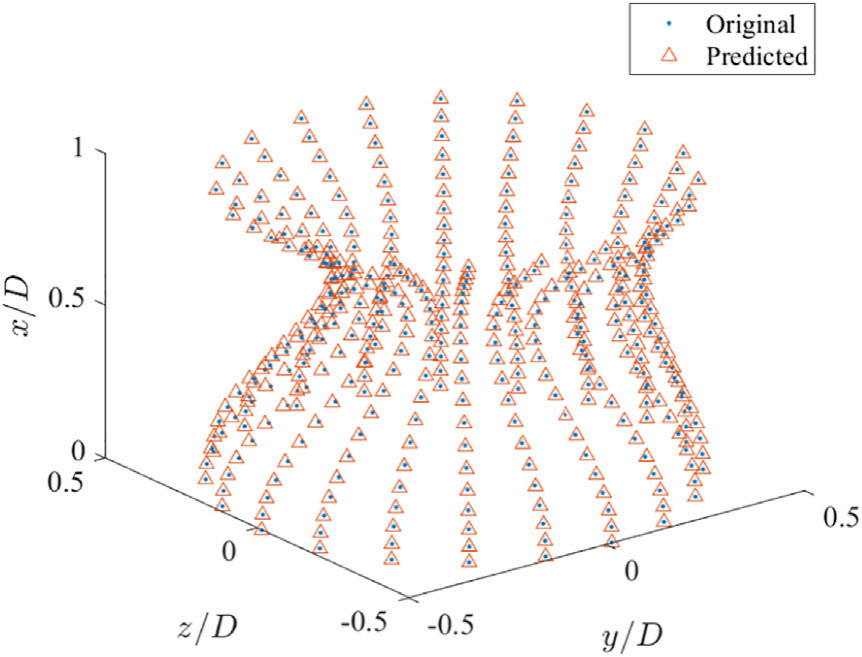
Comparison of the predicted and original coordinates of a 3D asymmetric stenosis.

As asymmetric arteries are considered, the established 2D CNN is not applicable. Here, a 3D CNN is further built to extract the stenosis features. The input of the 3D CNN is a 3D image with a dimension of 61 × 61 × 61, i.e., the resolution is 0.02*D*, which is generated by filling the pixels crossed by the stenosis as one with the rest as zero. The coordinates of 400 points evenly distributed on the stenosis surface are used as the output of the 3D CNN, with the mean-square-error as the loss function. ReLU and the hyperbolic tangent functions are used as the activation functions for the convolutional and fully connected layers, respectively. 800 examples are used as the training data with another 200 as the validation data. More details of the 3D CNN are shown in Table 2. Three numbers of the extracted features are tested, i.e., 8, 16 and 32, as an appropriate number of the stenosis feature can well represent the stenosis and improve the learning efficiency of the DNN. The histories of loss and mean absolute error versus epoch are shown in Figure 14. It is clear that all three networks show similar learning histories and good convergence, and the increase of features from 8 to 32 does not improve the accuracy. Therefore, 8 features are adopted for the further DNN training. An example to compare the predicted (by using 8 features) and original coordinates of 3D asymmetric stenosis is shown in Figure 15. It shows that the 3D CNN model predicts the coordinates well and thus has the ability to extract the features from complex stenosed arteries.

**TABLE 2:** Details of the 3D CNN.

Layer	Size	Filter size	Pooling filter	Output shape
Input	61 × 61 × 61 × 1	-	-	-
Conv1	32	4 × 4 × 4	3 × 3 × 3	61 × 61 × 61 × 32
Conv2	64	4 × 4 × 4	3 × 3 × 3	71 × 71 × 71 × 64
Conv3	128	4 × 4 × 4	3 × 3 × 3	23 × 23 × 23 × 128
Fully Connected × 2	128	-	-	128
Fully Connected (extracted features)	8/16/32	-	-	8/16/32
Fully Connected × 2	128	-	-	128
Fully Connected	-	-	-	800
Reshaped	-	-	-	400 × 2

**FIGURE 16:**
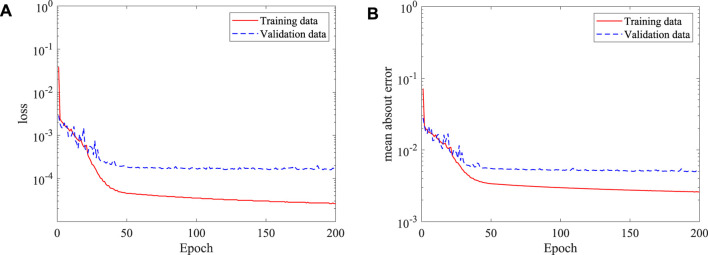
Histories of loss and mean absolute error of the DNN for asymmetric stenosis: **(A)** loss, and **(B)** mean absolute error.

**FIGURE 17:**
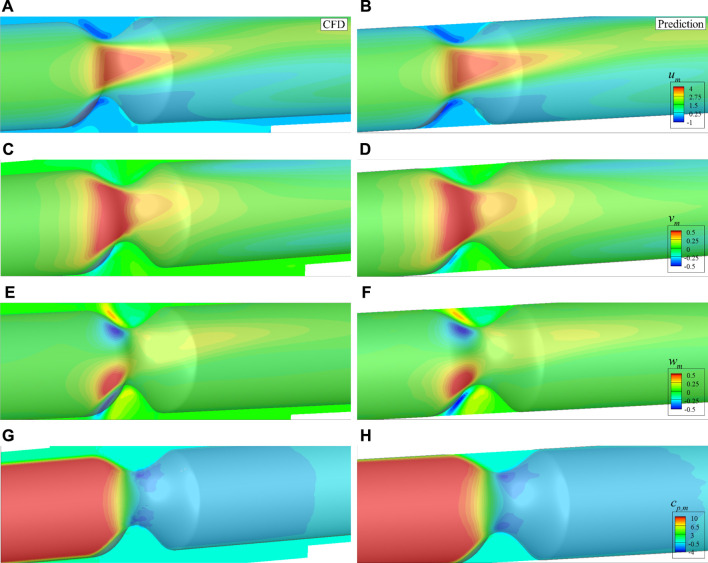
Comparison of the mean velocities and pressure with asymmetric stenosis on the plane of *z* = 0: **(A)** *u*_*m*_ by CFD, **(B)** *u*_*m*_ by DNN prediction, **(C)** *v*_*m*_ by CFD, **(D)** *v*_*m*_ by DNN prediction, **(E)** *w*_*m*_ by CFD, **(F)** *w*_*m*_ by DNN prediction, **(G)** *c*_*p*,*m*_ by CFD, **(H)** *c*_*p*,*m*_ by DNN prediction.

After the features of the stenosis are successfully extracted by the established 3D CNN, the DNN model built in the last section is adopted for the 3D asymmetric flow prediction. Similarly, 120 CFD simulations are conducted to obtain the training dataset, with 20 of them serving as the validation data. The histories of loss and mean absolute error are shown in Figure 16. It is found that the DNN converges after around 150 epochs, the mean absolute error (MAE) is less than 1%, which is much lower than the best value (6.2%) achieved by Liang et al. (2020) considering flow in human thoracic aorta. An example of the mean velocities and pressure fields obtained by the DNN and CFD simulation are shown in Figure 17. It is clear that the DNN gives an excellent prediction compared with the high fidelity CFD simulation in a significantly less expensive way. Almost all flow details such as the velocities and pressure change can be accurately captured.

The 2D and 3D stenosed arteries are considered, and the present results show that the combination of CNN for extracting stenosis features and the fully connected DNN for the prediction of blood flows works well in capturing the flow details in stenosed arteries. The success of this combination can be attributed to the versatility of CNN in extracting features of complex images (Badrinarayanan et al., 2017), which can be easily extended to arbitrarily complex stenosis once the required dataset is obtained. The computation time by using full CFD simulation and the DNN to predict the blood flow is further presented in Table 3 for comparison. It shows that the blood flow in stenosed arteries can be obtained in a few seconds for 2D and about 1 minute for 3D after the model is trained. The prediction time is in seconds excluding the time of initializing and file I/O, and it is comparable to that of other similar methods e.g., Ref. (Liang et al., 2020). In some medical applications, we only need to reconstruct the wall shear stress or the pressure drop due to the stenosis, which requires even less computational time. Therefore, the trained DNN method is significantly more efficient than the CFD simulation, and may have a great potential in clinic applications. It should be noted that more training sets may be required when more factors are included. Specifically, if the stenosis length is varied, the training set will not increase and the data size increases linearly. If the inlet velocity is varied, the training dataset will increase linearly and each data will be the same size. Therefore, the training dataset will increase linearly when we include two or more factors. New datasets may be required for cases with different topologies.

**TABLE 3:** Details of the computation time.

Type	Wall-time
CNN-2D training	24 cpu hours
CNN-3D training	912 cpu hours
DNN-2D training	80 cpu hours
DNN-3D axis-symmetric training	500 cpu hours
DNN-3D asymmetric training	960 cpu hours
CFD-2D	0.6 cpu hours per simulation
CFD-3D	50 cpu hours per simulation
DNN-2D prediction	6 s
DNN-3D prediction	65 s

## Conclusion

This paper has introduced a fast prediction method of blood flow in stenosed arteries with a hybrid framework of machine learning and IB–LBM which incorporates the immersed boundary method, the MRT LBM and the DNN, and takes advantages of their strengths. Several validation cases have been conducted by training and testing a DNN for the fast flow prediction in stenosed arteries with the CFD generated data. The results show that the DNN can predict the mean flow fields accurately with the results being available within about 1 minute, which is improved at least 1,000 times compared with direct numerical simulation. In addition to parametrise the stenosis by using an analytical polynomial, a 2D and a 3D CNNs are constructed to extract the stenosis features from 2D and 3D images, respectively. The results show that the CNN has a good performance in representing the stenosis and can be straightforwardly extended to arbitrarily complex stenosis, as demonstrated by the 3D asymmetric stenosis. Although the stenosis is generated by an analytical model in this work, the versatility of the CNN makes it applicable to patient-specific geometries that can be modelled with more parameters.

This work has successfully demonstrated the superiority to use machine learning and CFD based data for the fast prediction of mean flow fields in complex stenosed arteries. The future work will be focused on more realistic problems to include the non-Newtonian fluid, the pulsatile flow and more complex stenosis to obtain a more versatile DNN model.

## Data Availability

The raw data supporting the conclusions of this article will be made available by the authors, without undue reservation.
